# Shaping decision-making with screen time: video-based dialectical behavior therapy skills training for college students

**DOI:** 10.3389/fpsyg.2025.1609744

**Published:** 2025-07-09

**Authors:** Diana Mejía, Laurent Avila-Chauvet

**Affiliations:** Psychology Department, Sonora Institute of Technology, Obregón, Mexico

**Keywords:** decision-making, DBT, risk-taking, impulsivity, mindfulness, prosocial behavior

## Abstract

**Introduction:**

Dialectical Behavior Therapy (DBT) is a transdiagnostic treatment that can address various mental health issues across diverse populations, including college students. However, a knowledge gap persists regarding how DBT might influence cognitive processes, particularly decision-making, through a brief intervention.

**Methods:**

This study aimed to assess the effects of Video-DBT skills training on decision-making tasks among college students via an online platform. Ninety-five participants (comprising 50 men, 44 women, and one non-binary individual, age: *M* = 27.04, SD = 12.47) were randomly assigned to four core DBT skills groups. Participants received DBT training and underwent evaluation using three behavioral decision-making tasks: the Iowa Gambling Task (IGT), Delay Discounting (DD), and social discounting (SD).

**Results:**

Although intriguing trends in discounting rates and IGT performance were observed among various groups before and after training, no statistically significant differences were found either among the groups or within individual groups. Nevertheless, it was discovered that distress tolerance and interpersonal effectiveness skills showed promise in reducing impulsivity and enhancing prosocial behavior.

**Discussion:**

The findings highlight the potential of DBT skills training to influence cognitive and behavioral outcomes related to decision-making, even in the context of a brief teleintervention. While no statistically significant differences were observed, trends in discounting rates and IGT performance suggest that certain DBT skills, particularly distress tolerance and interpersonal effectiveness, may play a role in reducing impulsivity and fostering prosocial behavior.

## 1 Introduction

Attending college is a pivotal phase in the lives of young adults, often perceived as a time of personal growth, intellectual exploration, and the pursuit of academic excellence. However, this transitional period is challenging, as college students contend with various stressors that can significantly impact their mental health and wellbeing. Pedrelli et al. (2015) highlighted the stressors college students face, ranging from the rigors of academic coursework to the complexities of separating from their family of origin and juggling various work and family responsibilities. In this context, it is unsurprising that many college students experience the onset or exacerbation of mental health and substance use problems, undermining their overall quality of life.

Indeed, carefree college years belies a stark reality: nearly half of college students can be diagnosed with at least one mental health disorder within a year (Blanco et al., 2008). Depression, suicidal ideation, non-suicidal self-injury (NSSI), and borderline personality disorder (BPD) represent significant mental health challenges among this demographic. Surprisingly, approximately one-third of college students report experiencing depression that has negatively impacted their ability to function in the past year (American College Health Association, 2012).

Considering the commonality and severity of mental health within the college student population, it is important to explore innovative approaches to help mitigate their adverse effects. Dialectical Behavior Therapy (DBT), initially developed to treat borderline personality disorder, has shown promise in addressing emotional dysregulation, impulsivity, and interpersonal difficulties—core challenges experienced by college students. DBT is also a transdiagnostic treatment, meaning it can address various mental health issues among different populations, including college students (Chugani, 2015, 2017). One study explored an adaptation of DBT tailored to address the specific needs of college students diagnosed with ADHD; Students reported improvements in quality of life, use of mindful nonjudgement skills, and the number of ADHD symptoms decreased (Weathers, 2022). Similarly, the program DBT STEPS-A, a school-based adaptation for Mexican college students, showed statistically significant improvement in depression and anxiety symptoms (Hernández et al., 2021). Even short-term interventions such as DBT mindfulness and distress tolerance skills can Benefit college students (Muhomba et al., 2017).

DBT in college settings seamlessly integrates with virtual technology, bridging therapeutic innovation with modern-day accessibility for students. The inclusion of digital platforms in Dialectical Behavior Therapy (DBT) is not just about accessibility but also concerns the efficacy and user experience. One study compared telehealth and in-person group therapy, specifically in a DBT-based intensive outpatient program IOP. The results of this study contribute to our comprehension of the concrete effects of telehealth platforms, In both groups, there was a large reduction in symptoms following completion of the IOP for both the in-person and videoconference groups (Bean et al., 2022). Nonetheless, a review of articles concludes that there is a necessity to conduct comparisons between standard DBT and its online counterpart and blended DBT and consider the use of smartphone apps and virtual reality (VR) as facilitators in access and implantation of DBT skills (van Leeuwen et al., 2021). Besides, the effectiveness of an online platform is not merely about replicating in-person therapy but optimizing the digital experience to retain participants. If users find the platform cumbersome or non-intuitive, the dropout rates can be significantly higher, compromising the potential therapeutic benefits (Wilks et al., 2020).

However, there remains a gap in our understanding of how DBT may impact cognitive processes, specifically decision-making using an online platform. This study aims to bridge this gap by investigating the effects of DBT on decision-making tasks among college students through an online platform. We want to focus on how DBT may influence impulsivity and risk-taking, traits closely associated with various mental health challenges (Bornovalova et al., 2005; Chan et al., 2023). To evaluate the impact of DBT, we used two behavioral tasks: Delay Discounting and Iowa Gambling Task (IGT), which are recognized measures for assessing impulsivity (Acuff et al., 2023; Kluwe-Schiavon et al., 2020; Mejía et al., 2022). Regarding social decision-making, the most common behavioral task is social discounting. The performance in this task has been related to prosocial behavior. Some studies have found that clinical populations (e.g., individuals with substance use disorders, autism, or externalizing behavior problems) tend to demonstrate a lower propensity to share resources or engage in prosocial behavior compared to control participants (Avila-Chauvet et al., 2023; Bradstreet et al., 2012; Sharp et al., 2012; Warnell et al., 2019).

Delay discounting tasks assess an individual’s willingness to forgo a smaller, immediate reward instead of a larger, delayed one. High impulsivity is often linked to a preference for smaller, more immediate rewards, reflecting an inability to delay gratification. Social discounting tasks examine how individuals make decisions regarding the allocation of resources, particularly in social contexts. Impulsivity can manifest in a tendency to choose immediate self-interest over longer-term considerations within social interactions. The Iowa Gambling Task assesses decision-making under ambiguity and risk, where individuals with higher impulsivity may exhibit poorer decision-making strategies, leading to financial losses. Previous studies have used behavioral tasks to measure the effects of DBT skills on impulsivity tasks in a clinical population, where the training improved their ability to delay gratification, time perception, and decision-making with IGT (Cavicchioli et al., 2023; Soler et al., 2016).

Research has demonstrated that delay discounting on monetary earnings remains consistent following cognitive-behavioral treatment CBT, which includes mindfulness training and contingency management (MC), for individuals using tobacco (López, 2014; López-Torrecillas et al., 2014; Secades-Villa et al., 2014; Weidberg et al., 2015), alcohol (Dennhardt et al., 2015; De Wilde et al., 2013; Jones, 2013), and other illicit drugs (Weidel, 2013; Mejía et al., 2016). However, Landes et al. (2012) study on users with opioid dependence showed varied results: 48% of the sample maintained their discount rates, 39% exhibited a decrease, and 12% experienced an increase. Notably, these changes showed no correlation with the specific treatment received. Contrary to CBT and MC treatments, studies have indicated that training in working memory reduces the temporary discount rates of monetary gains among stimulant users (Bickel et al., 2011). Additionally, the Acceptance-Based Procedure has been shown to be an effective intervention for reducing delay discounting rates. A single session lasting between 60 and 90 min significantly decreased discounting among healthy college students (Morrison et al., 2014). This study highlights the importance of incorporating evidence from brief interventions and underscores their potential cost-effectiveness in implementation.

Understanding how Dialectical Behavior Therapy (DBT) influences decision-making tasks may offer critical insights into the therapy’s effectiveness in enhancing cognitive processes and self-regulation among college students. By examining these behavioral measures alongside mental health outcomes, we aim to provide valuable evidence to inform the development of targeted interventions for this population, as well as to deepen our understanding of the complex interplay between telehealth delivery, DBT skills, and decision-making during this pivotal stage of life. Brief interventions, in particular, could be feasibly integrated into college curricula and offer a cost-effective alternative to more intensive, long-term treatments.

We hypothesize that the four core DBT skills—Mindfulness, Emotion Regulation, Distress Tolerance, and Interpersonal Effectiveness—will enhance performance by leading participants to select more advantageous cards in the IGT task. Additionally, we expect these skills will decrease delay and social discounting rates, thereby reflecting reduced impulsivity and heightened prosocial behavior.

## 2 Method

### 2.1 Participants

In our study, we recruited healthy college students from southern Sonora, México, using a snowball sampling method. Participants were offered extra credit in psychology-related courses as an incentive for their participation. We initially began with 133 participants, but our analysis focused on 95 participants (50 men, 44 women, and one non-binary, age: *M* = 27.04, *SD* = 12.47). These 95 participants demonstrated a sufficient level of engagement with behavioral tasks and video skills training, as indicated by their scores, see details in procedure.

Eligibility for the study required participants to be above 18 years of age, and to disclose their history of substance use. Following the ethical principles for human medical research outlined in the Declaration of Helsinki, informed consent was obtained from all participants. The research protocol received approval from the Sonora Institute of Technology’s Institutional Review Board (ID 224).

### 2.2 Instruments

We developed a digital platform compatible with Android to facilitate behavioral tasks, administer questionnaires, and provide video training. Some tasks integrated into the platform include the Iowa Gambling Task (IGT), Delay Discounting (DD), and social discounting (SD). Each task is linked to specific DBT group skills and can be accessed via the following URLs (see Supplementary material):

Mindfulness: http://lcaa.com.mx/DBTD/M/; Interpersonal Effectiveness: http://lcaa.com.mx/DBTD/E/; Emotion Regulation: http://lcaa.com.mx/DBTD/R/; Distress Tolerance: http://lcaa.com.mx/DBTD/T/; Documentary Video on Vivaldi’s Music (Control): http://lcaa.com.mx/DBTD/C/.

In addition, we have incorporated two questionnaires through Google Forms. The first collects demographic information, including gender, age, income, dietary habits, life satisfaction scale, sleeping patterns, and alcohol, tobacco, and other illicit substances consumption. The second questionnaire evaluates the user’s knowledge about various DBT skill groups.

The Iowa Gambling Task (IGT) is a behavioral task designed to assess decision-making. The participants are given four decks of cards that differ in wins, losses, and the probability of loss. Over time, choosing from decks with smaller gains and smaller losses becomes advantageous, as these decks pose less risk. Conversely, while decks with higher gains might appear beneficial in the short term, they have the potential for significant losses. The primary metric for analysis is the proportion of advantageous selections made in each block, where a value of 1 indicates a predominantly advantageous choice, and 0 indicates a predominantly disadvantageous choice. The test comprises 100 trials (Bechara et al., 1994). Performance on the Iowa Gambling Task (IGT) has been correlated with impulsivity scales (Buelow and Blaine, 2015). Regarding test–retest reliability, the IGT demonstrated no significant correlations between performance on Trials 1–40 at Time 1 and Time 2. However, weak correlations were observed between performance on Trials 41–100 at Time 1 and Time 2. Paired-samples t-tests for both sets of trials (1–40: *t*(93) = −4.50, *p* = 0.001, *d* = 0.47; 41–100: *t*(93) = −2.98, *p* = 0.004, *d* = 0.31) indicated a significantly lower risk-taking tendency at Time 2 compared to Time 1 (Buelow and Barnhart, 2018).

The Delay Discounting (DD) task assesses the effects of delay on the subjective value of a reward before its receipt. In this task, participants are shown two choices on a screen: a small immediate reward or a large delayed one. Impulsivity is inferred when a participant chooses immediate small rewards instead of waiting for larger delayed rewards. Our methodology employed an adjusting-amount procedure over seven delays (7 days, 30 days, 180 days, 365 days, and 1,095 days). The primary metric for evaluation was the Area Under the Discounting Curve (AUC). Here, a score of 0.0 represents the highest possible degree of impulsivity, while 1.0 signifies maximum self-control. For a detailed explanation of this metric, see Myerson et al. (2001). To estimate participants’ discounting rate, we fit the hyperbolic equation *V* = *A*/1 + *bx* where *V* is the subjective value of the delayed outcome, *b* is the discount rate, and *D* is the delay. Higher *b* values are interpreted as impulsivity. Regarding validity and reliability, performance on delay discounting (DD) tasks has been associated with impulsivity and substance use disorders (Acuff et al., 2023). The DD task has demonstrated substantial test–retest correlations (*r* = 0.67 and 0.76, *p* < 0.001, 95% CI [0.618, 0.716]) and minimal changes in effect magnitude over time (*dz* = 0.048, *p* = 0.31, 95% CI [−0.051, 0.146]) (Anokhin et al., 2015).

The Social Discounting Task (SD) examines how social distance influences the perceived paramount to the participant, while the 100th is someone they barely know, perhaps someone who does not even remember their name (Jones and Rachlin, 2009). The social discounting task has demonstrated strong test–retest correlations in both current smokers (*r* = 0.85) and non-smokers (*r* = 0.82) (Tuen et al., 2023). Additionally, current smokers tend to exhibit steeper social discounting compared to non-smokers (Wainwright et al., 2018).

The participants choose between taking this money for themselves or giving it to a member of the list, occupying position x. Initially, for each individual on the list, participants face a choice: share $200 with that person or keep $100 for themselves. If participants choose to keep the $100, the alternative’s value drops by 50% in the next trial (down to $50). Conversely, if they decide to share the $200 with the person from the list, their potential self-reward in the next trial rises by 50% (to $150). This process is repeated in seven trials for six positions from the list (1, 2, 10, 20, 50, and 100), chosen randomly, and evaluated in the trials. The Area Under the Discounting Curve (AUC), and the *b* value parameter of the hyperbolic equation allow us to estimate how each participant discounted the monetary consequence as the social distance rises. Higher *b* values and a score close to 0.0 in AuC are interpreted as less altruistic.

Questionnaire of DBT skills: We developed four questionnaires for each group of DBT skills. Each questionnaire had five questions related to the video training. The highest possible score was 5 points on each questionnaire. In the sample analyzed, we chose the participants with scores between 3 and 5.

Life satisfaction scale. The instrument comprises 10 items that assess an individual’s level of life satisfaction on a scale from 0 to 10, spanning nine specific areas of their daily life and overall satisfaction. Its reliability coefficient stands at *α* 0.76 (Barragán Torres et al., 2005). The average among the 10 items was the dependent variable. Scores close to 10 indicate greater life satisfaction.

### 2.3 Procedure

The training was evaluated using a randomized controlled design to compare the performance of five groups across three behavioral tasks against a control condition. Participants were informed that their participation would earn them extra credit in psychology-related courses. The evaluation took place from July 2022 to April 2023, with each session lasting approximately 90 min on average.

Participants were invited to a computer lab at the college to complete the evaluation. Prior to starting, each participant was randomly assigned to one of five conditions and provided with a unique code, which they used to access their assigned tasks on the platform. Participants were also asked to complete a Google Form to provide demographic information (e.g., approximate personal monthly income in pesos; if there was no income, they were instructed to enter zero) and to indicate their informed consent to participate. Details of these questions are available in Supplementary material. They were then directed to access the platform via specific links corresponding to their assigned group and complete the following behavioral tasks: the Iowa Gambling Task (IGT), Delay Discounting Task (DD), and Digit Span Task (DS). Assistance was available during the tasks for any questions or technical issues.

After the initial evaluation, participants viewed a 20-min training video associated with their assigned group. They were allowed to watch the video up to three additional times to address any questions or reinforce their understanding. Upon completing a questionnaire about DBT (Dialectical Behavior Therapy) skills, participants proceeded to complete the behavioral tasks (IGT, DD, and DS) a second time. Upon completion, participants received a certificate confirming their participation, which they could use for extra credit.

*Video-Based DBT Skills Training.* The DBT skills were segmented into four distinct groups, as outlined in the DBT Skills Training Manual (Linehan, 2015a,b). Each instructional video was approximately 20 min long and covered the following key areas:

For Mindfulness, the skills explained were in the following order: “What” Skills: observe, describe, participate; “How” Skills: nonjudgmentally, one-mindfully, effectively; Balancing doing mind and being mind; Ideas for Practicing Wise Mind: Stone flake on the lake; Walking down the spiral stairs; Breathing “Wise” in, “Mind” out; Asking Wise Mind a question.

For Interpersonal Effectiveness, the skills explained were in the following order: Goals of Interpersonal Effectiveness; Myths in the Way of Interpersonal Effectiveness; Applying DEAR MAN Skills to a Difficult Current Interaction; Guidelines for Relationship Effectiveness: Keeping the Relationship (GIVE); Guidelines for Self-Respect Effectiveness: Keeping Respect for Yourself (FAST).

In Emotion regulation, the skills explained were in the following order: Understanding and Naming Emotions; What Emotions Do for You; Myths about Emotions; Check the facts, Opposite Action, Problem solving; Building a Life Worth Living, ABC skills, and PLEASE skills.

For Distress Tolerance, the skills explained were in the following order: STOP skill; Pros and Cons skill; TIP Your Body Chemistry; Distract with Wise Mind ACCEPTS; Self-Soothe with the Five Senses; Improve the Moment.

Each section provides a comprehensive overview of the respective skills and strategies, facilitating a deeper understanding and application of DBT techniques. We provided examples of situations where they can apply the skills. See details of each group of skills in the DBT Skills Training Manual (Linehan, 2015a,b), and DBT skills training handouts and worksheets (Linehan, 2015a,b).

The control group viewed a 20-min documentary video on Vivaldi’s music, which provided an overview of the composer’s life, key works, and historical context. Following the video, participants completed a questionnaire designed to assess their comprehension and retention of the information presented in the documentary. This task served as a neutral activity to control for the time and cognitive engagement associated with the experimental group’s training sessions.

### 2.4 Data analysis

We calculated the frequencies, means, and standard deviations for the dependent variables of each behavioral task, as well as for the participants’ demographic characteristics. Normality analyses were conducted to determine whether to use parametric or non-parametric tests, including the Kolmogorov–Smirnov and Levene tests, to assess the homogeneity of variances for each independent variable. Normality tests indicated that non-parametric tests were appropriate. A Kruskal-Wallis test to contrast the five groups was used in each behavioral task performance pre-training and post-training. We used the Kruskal-Wallis’s test to contrast demographic characteristics among the five groups. We used the Wilcoxon Signed Rank Test to test the differences between the pre-test and post-test for each group.

To employ the hyperboloid function in modeling the delay discounting task, we utilized the equation: *V* = 1/(1 + *bX*^s^). In this equation, “*V*” represents the subjective value assigned to the delayed outcome, “*b*” is a parameter indicating the rate at which the subjective value decreases as the delay to receive the outcome increases (whether due to temporal delay or greater social distance), and “*X*” denotes the measure of delay or social distance (Mejía et al., 2022).

In our analysis of participants’ performance in the Iowa Gambling Task (IGT), we employed a power function (*Y* = *ax^b^*). Higher values of “*b*” within this context denote an augmented preference for advantageous alternatives across the task’s successive blocks (Mejía et al., 2022).

The correlations among decision-making measures were explored using Pearson correlations for all participants. We also measured the correlations between demographic characteristics, substance use, and life satisfaction. All statistical analyses were carried out in the JASP v0.17.1® program.

## 3 Results

### 3.1 Group characteristics

There were no significant differences among the groups concerning age (*p* = 0.723), years of education (*p* = 0.115), monthly income (*p* = 0.214), alcohol consumption (*p* = 0.590), the number of tobacco cigarettes (*p* = 0.346), illegal drug use (*p* = 0.249), the number of marijuana cigarettes (*p* = 0.097), hours of sleep (*p* = 0.918), and life satisfaction (*p* = 0.332) (see Table 1).

**Table 1:** Sociodemographic characteristics.

	Controls		Interpersonal effectiveness		Mindfulness		Emotion regulation		Distress tolerance			
*N*	25		17		25		12		16			
	*M*	SD	*M*	SD	*M*	SD	*M*	SD	*M*	SD	*X^2^*	*p*
Age	25.32	11.23	30.05	14.31	26.20	12.12	31.00	14.56	24.87	11.35	2.06	0.723
Years of the study	15.08	1.99	13.47	3.10	14.16	2.89	15.08	1.37	14.43	1.82	7.42	0.115
Monthly Income	$3,322	$5,510	$1,326	$2,093	$1,728	$2,356	$3,825	$4,160	$3,525	$3,883	5.80	0.214
Number of drinks	2.24	3.01	1.52	2.60	1.88	2.55	1.00	1.414	2.75	3.19	2.78	0.59
Number of Illegal drugs by week	0.040	0.20	0.0	0.0	0.120	0.440	0.167	0.389	0.250	0.557	5.39	0.249
Number of tobacco cigarette	0.200	0.816	0.059	0.243	0.400	1.041	0.083	0.289	1.563	3.86	4.47	0.346
Number marijuana cigarette	0.040	0.200	0.0	0.0	0.080	0.277	0.167	0.577	0.313	0.602	7.84	0.097
Hours of sleep	7.08	1.15	7.05	1.08	6.84	1.37	6.83	1.030	6.62	1.44	0.94	0.918
Life satisfaction	6.84	1.71	7.74	1.96	6.79	2.18	6.38	1.88	6.33	2.28	4.58	0.332

### 3.2 Results of delay discounting task

**Figure 1:**
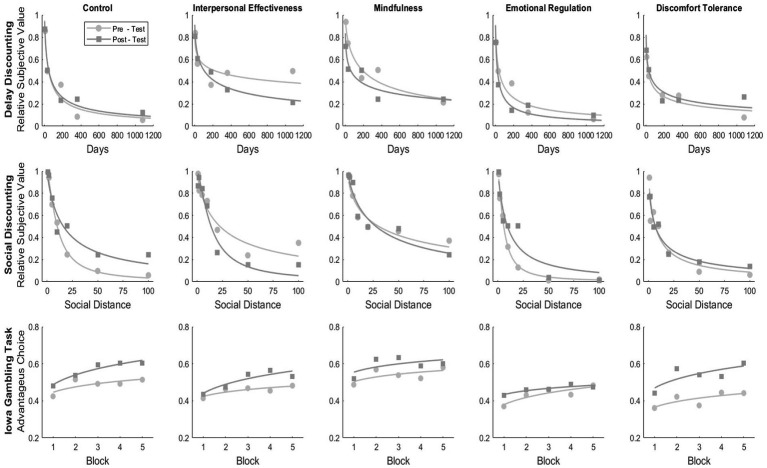
Fitting hyperbolic model to social and temporal discounting curves, and power function fitting to Iowa gambling task blocks.

Table 2 and Figure 1 show, by group, the discounting rate for delay discounting, social discounting tasks, and the proportion of advantageous alternative choices in 20-card blocks in the Iowa Gambling Task. The results in the first row of Figure 1 show the changes in the delay discounting rate across different groups before and after training. For the Control, Mindfulness, and Emotional Regulation groups, the discount rate (*k*) increased and exhibited good fit values over 0.85. While in the case of the Interpersonal Effectiveness and Discomfort Tolerance groups, the discount rate decreased after treatment, suggesting a reduction of the preference for smaller immediate alternatives or impulsivity. However, a Kruskal-Wallis Test indicates that there were no statistically significant differences in the discounting rate between the groups in the pre-test (*X^2^* = 6.64, *p* = 0.38) and post-test (*X^2^* = 2.20, *p* = 0.69) (see Table 2). On the other hand, Wilcoxon Signed Rank Test reveals no significant differences between the pre-test and post-test in control (*W* = 68.0, *p* = 0.06), Interpersonal Effectiveness (*W* = 84.0, *p* = 0.74), Mindfulness (*W* = 90.0, *p* = 0.052), Emotional Regulation (*W* = 58.0, *p* = 0.15), Discomfort Tolerance (*W* = 61.0, *p* = 0.97) groups.

**Table 2:** Parameters from the hyperboloid function fitted to social and delay discounting curves, and from the power function fitted to Iowa Gambling Task performance across blocks.

	Delay discounting						Social discounting						IGT			
	Pre-test			Post-test			Pre-test			Post-test			Pre-test		Post-test	
	*b*	*s*	*R^2^*	*b*	*s*	*R^2^*	*b*	*s*	*R^2^*	*b*	*s*	*R^2^*	*b*	*R^2^*	*b*	*R^2^*
Control	0.05	0.78	0.93	0.06	0.72	0.95	0.03	1.46	0.99	0.08	0.90	0.92	0.09	0.58	0.15	0.94
Interpersonal Effectiveness	0.23	0.28	0.59	0.10	0.51	0.97	0.08	0.81	0.91	0.02	1.44	0.95	0.08	0.73	0.15	0.81
Mindfulness	0.03	0.64	0.93	0.20	0.40	0.86	0.10	0.66	0.91	0.06	0.83	0.92	0.07	0.41	0.07	0.36
Emotional Regulation	0.10	0.64	0.93	0.11	0.73	0.94	0.08	1.45	0.99	0.10	1.02	0.91	0.14	0.81	0.07	0.82
Discomfort Tolerance	0.25	0.46	0.95	0.22	0.45	0.90	0.19	0.88	0.89	0.24	0.74	0.95	0.11	0.57	0.14	0.60

*b* represents the discounting rate; s is a nonlinear scaling or weighting parameter; *R*² indicates the proportion of variance explained by the model fit; and *b* (in the context of the Iowa Gambling Task) denotes the rate of preference for advantageous alternatives.

**Figure 2:**
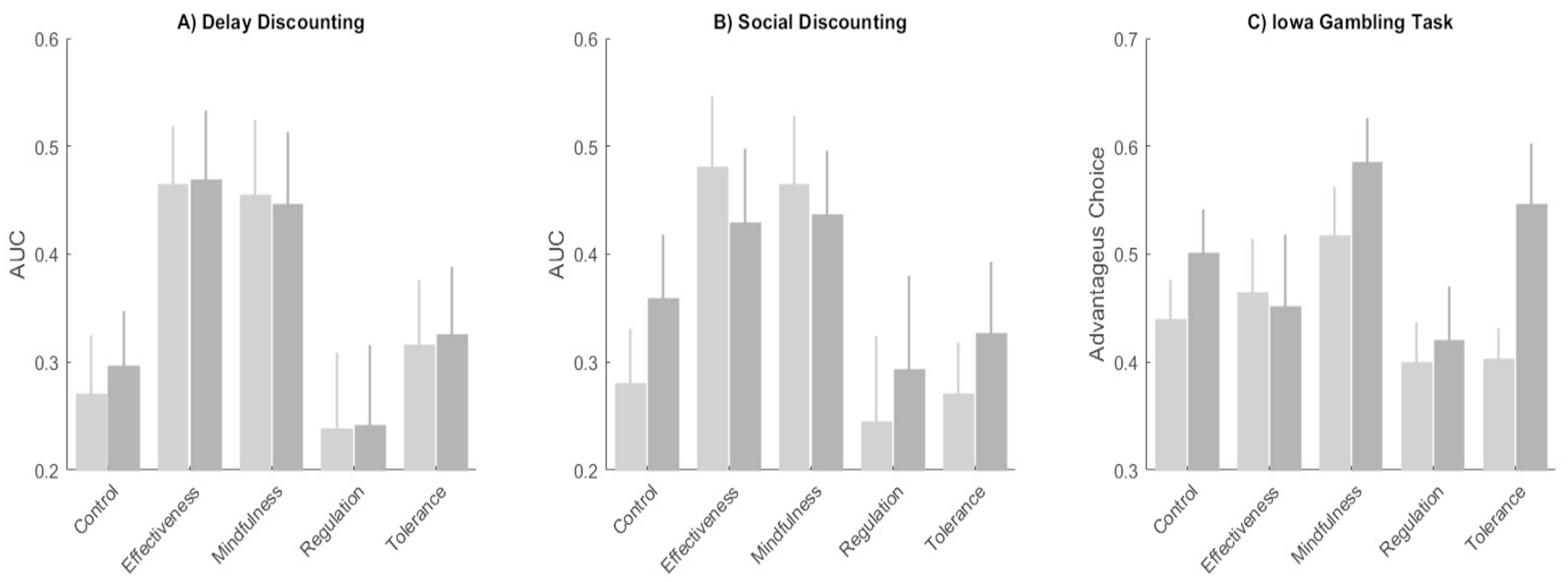
Area under the curve for social and delay discounting and relative preference for advantageous choices in gambling task blocks.

Figure 2 shows, by group, the area under the curve for delay and social discounting tasks and the relative preference for advantageous alternatives in the Iowa Gambling Task. Figure 1A shows the average area under the curve in the delay discounting task by group. A Kruskal-Wallis Test indicates that there were significant differences in the AUC between the groups in the pre-test (*X^2^* = 10.28, *p* = 0.04) but not in the post-test (*X^2^* = 7.95, *p* = 0.09). Wilcoxon Signed Rank Test reveals no significant differences between the pre-test and post-test in control (*W* = 89.5, *p* = 0.97), Interpersonal Effectiveness (*W* = 74.0, *p* = 0.92), Mindfulness (*W* = 128.0, *p* = 0.77), Emotional Regulation (*W* = 39.5, *p* = 0.10), and Discomfort Tolerance (*W* = 55.0, *p* = 0.79) groups.

### 3.3 Results of social discounting task

The second row of Figure 1 shows the changes in the social discounting rate across different groups before and after training. For the Control, Emotional Regulation and Discomfort Tolerance groups, the discount rate (*k*) increased and exhibited good fit values over 0.90. While in the case of the Interpersonal Effectiveness and Mindfulness groups, the discounting rate decreased after treatment, suggesting a decrease in social distance or an increase in altruism (see Table 3). However, a Kruskal-Wallis Test indicates that there were no statistically significant differences in the discounting rate among the groups in the pre-test (*X^2^* = 3.72, *p* = 0.44) and post-test (*X^2^* = 5.01, *p* = 0.28). Wilcoxon Signed Rank Test reveals no significant differences between the pre-test and post-test in control (*W* = 182.0, *p* = 0.36), Interpersonal Effectiveness (*W* = 46.0, *p* = 0.26), Mindfulness (*W* = 118.0, *p* = 0.36), Emotional Regulation (*W* = 15.0, *p* = 0.12), Discomfort Tolerance (*W* = 51.0, *p* = 0.40) groups.

**Table 3:** Means and standard deviations of the decision-making tasks.

	Controls		Interpersonal effectiveness		Mindfulness		Emotion regulation		Distress tolerance				
*N*	25		17		25		12		16				
	*M*	SD	*M*	SD	*M*	SD	*M*	SD	*M*	SD	*X^2^*	*p*	*d*
Pre training													
AuC delay discounting	0.269	0.278	0.464	0.228	0.454	0.350	0.238	0.247	0.315	0.245	10.28	0.040	0.103
AuC social discounting	0.279	0.259	0.479	0.275	0.464	0.320	0.244	0.277	0.269	0.196	11.58	0.021	0.126
Advantage cards IGT	0.439	0.189	0.463	0.212	0.516	0.229	0.399	0.131	0.402	0.117	1.964	0.742	0.054
Post training													
AuC delay discounting	0.295	0.260	0.468	0.269	0.445	0.342	0.241	0.260	0.324	0.256	7.95	0.093	0.08
AuC social discounting	0.358	0.299	0.428	0.289	0.436	0.300	0.292	0.303	0.326	0.270	3.68	0.451	0.033
Advantage cards IGT	0.500	0.210	0.451	0.279	0.84	0.211	0.419	0.177	0.545	0.231	6.212	0.184	0.066

IGT, Iowa Gambling Task, average of advantageous selection by 20-trials blocks; Area Under the Curve (AUC) of delay discounting; AUC of social discounting.

Figure 2 shows the average area under the curve in the social discounting task. A Kruskal-Wallis Test indicates that there were significant differences in the AUC between the groups in the pre-test (*X^2^* = 11.58, *p* = 0.02) but not in the post-test (*X^2^* = 3.68, *p* = 0.45). Wilcoxon Signed Rank Test show significant differences between the pre-test and post-test in control (*W* = 42.5, *p* = 0.01) but not Interpersonal Effectiveness (*W* = 76.0, *p* = 0.69), Mindfulness (*W* = 136.0, *p* = 0.48), Emotional Regulation (*W* = 14.0, *p* = 0.10), and Discomfort Tolerance (*W* = 37.0, *p* = 0.21).

### 3.4 Results of Iowa gambling test

The third row of Figure 1 shows the relative preference for advantageous alternatives across 20-card blocks for the different groups before and after treatment. The parameter ‘*b*’ which indicates the change in preference for alternatives or the transition from uncertainty to risk situation, increased for the control, Interpersonal Effectiveness, and Discomfort Tolerance groups, suggesting a progressive increase in preference for advantageous alternatives. However, a Kruskal-Wallis Test indicates that there were no statistically significant differences between the groups in the pre-test (*X^2^* = 0.78, *p* = 0.34) and post-test (*X^2^* = 3.78, *p* = 0.43) (see Table 3). Wilcoxon Signed Rank Test reveals no significant differences between the pre-test and post-test in control (*W* = 395.0, *p* = 0.48), Interpersonal Effectiveness (*W* = 115.0, *p* = 0.72), Mindfulness (*W* = 277.0, *p* = 0.57), Emotional Regulation (*W* = 75.0, *p* = 0.42), Discomfort Tolerance (*W* = 72.0, *p* = 0.58) groups.

Considering the advantage choice proportion, a Kruskal-Wallis Test indicates indicated there were no significant differences in the relative preference for advantageous choices among the groups in the pre-test (*X*^2^ = 1.96, *p* = 0.74) and post-test (*X*^2^ = 6.21, *p* = 0.18). Wilcoxon Signed Rank Test reveals significant differences between the pre-test and post-test in Discomfort Tolerance (*W* = 16.0, *p* = 0.005), but not in Interpersonal Effectiveness (*W* = 73.5, *p* = 0.79), Mindfulness (*W* = 74.5, *p* = 0.09), Emotional Regulation (*W* = 30.0, *p* = 0.51), and Control (*W* = 124.0, *p* = 0.30) groups.

### 3.5 Correlation analysis

Table 4 presents the correlation matrix for the pre-test results. Notably, a highly significant positive correlation is observed between the AUC (Area Under the Curve) of the Delay and Social discounting tasks (*p* < 0.001). This finding suggests a potential interaction between delay and social decision-making processes. Furthermore, correlations are identified between the AUC of discounting tasks and the Life Satisfaction Scale (Delay: *p* = 0.01, Social: *p* = 0.01). This implies that lower impulsivity and greater altruism contribute to overall life satisfaction.

**Table 4:** Correlation between the results of the tasks in the pretest.

	Delay AUC	Delay k	Social AUC	Social k	IGT V.	IGT b	LSS
1. Delay AUC	–						
2. Delay *k*	−0.182	–					
3. Social AUC	0.632***	−0.190	–				
4. Social *k*	−0.254*	0.553***	−0.269**	–			
5. IGT V.	0.138	0.049	0.011	−0.114	–		
6. IGT *b*	0.153	−0.010	0.121	−0.056	0.014	–	
7. LSS	0.245*	−0.235*	0.242*	−0.261*	0.119	−0.087	–

Bold values indicate statistically significant correlations among the variables. *p < 0.05, **p < 0.01, ***p < 0.001.

Interestingly, the IGT task does not exhibit significant correlations with the other behavioral tasks or the Life Satisfaction Scale, as indicated by *p* values > 0.5. When examining demographic variables, no significant correlations are found between these variables and the behavioral tasks, or among the demographic variables themselves, all of which have *p* values > 0.5.

## 4 Discussion

This study evaluated the effect of Video-Based DBT Skills Training for College Students on performing three behavioral tasks (pre-training and post-training). The discounting rate “*k*” showed varying trends before and after training in different groups. The Control, Mindfulness, and Emotional Regulation groups exhibited increased discounting rates, indicating a higher preference for smaller immediate alternatives or impulsivity. In contrast, the Interpersonal Effectiveness and Discomfort Tolerance groups displayed reduced discount rates after treatment, suggesting a reduction in impulsivity. However, it is essential to highlight that there were no statistically significant differences in discounting rates among the groups in both the pre-test and post-test. Additionally, the Wilcoxon Signed Rank Test revealed no significant differences between the pre-test and post-test within individual groups, except for a marginally significant change in the Control group (*p* = 0.06). We did not expect these results, we had hypothesized that all DBT skills would rise AuC and decrease *k* values, similar to Morrison et al. (2014), and Bickel et al. (2011) studies, especially with mindfulness skills. Nevertheless, in this community sample, we found similar results according to studies with clinical samples (Dennhardt et al., 2015; De Wilde et al., 2013; Jones, 2013; López, 2014; López-Torrecillas et al., 2014; Mejía et al., 2016; Secades-Villa et al., 2014; Weidberg et al., 2015).

In the Social Discounting Task, similar to the delay discounting task, varying trends in discounting rates were observed across different groups before and after training. Control, Emotional Regulation, and Discomfort Tolerance groups displayed increased discounting rates, indicating a preference for smaller immediate alternatives. In contrast, Interpersonal Effectiveness and Mindfulness groups exhibited decreased discounting rates, suggesting decreased social distance or increased altruism. However, similar to the delay discounting task, no statistically significant differences in discounting rates were found between the pre-test and post-test groups. The Wilcoxon Signed Rank Test also showed no significant differences between the pre-test and post-test within individual groups, except for a significant change in the Control group (*p* = 0.01). Like delay discounting, we expected a rise in AuC and decreased *k* values. Our study is one of the few studies that have explored how to modify social discounting rates. Although we did not find statistically significant differences, we found the effect of interpersonal effectiveness and Mindfulness. These skills tend to reduce social discounting rates (rise in prosocial behavior). In this group of skills, the verbal instructions display the importance of validating others, to understand that suffering is part of several human beings. This concept is similar to the finding of the study of Tuen et al. (2023) where they found that greater identification with all of humanity predicted shallower social discounting.

The Iowa Gambling Test (IGT) results specifically focus on the relative preference for advantageous alternatives across 20-card blocks. The parameter “*b*” representing the transition from uncertainty to risk situations, increased for the Control, Interpersonal Effectiveness, and Distress Tolerance groups, suggesting a progressive increase in preference for advantageous alternatives. However, similar to the discounting tasks, no statistically significant differences in “*b*” values were observed between the pre-test and post-test groups. The Wilcoxon Signed Rank Test also revealed no significant differences between the pre-test and post-test within individual groups. When considering the proportion of advantageous choices in the IGT, the Kruskal-Wallis Test showed no significant differences between groups in both the pre-test and post-test. The Wilcoxon Signed Rank Test revealed significant differences in the Distress Tolerance group between the pre-test and post-test, suggesting that this group exhibited changes in their preferences for advantageous choices. We expected more improvement in the task performance with all DBT skills. Nevertheless, we have similar results considering the group of skills with distress tolerance to previous research with clinical samples (Cavicchioli et al., 2023; Soler et al., 2016). It is also important to consider potential learning effects associated with the IGT. Although prior evidence has shown no significant correlations between performance on Trials 1–40 at Time 1 and Time 2, weak correlations were observed for Trials 41–100 across the same time points (Buelow and Barnhart, 2018).

In order to complement the primary analyses and enhance the robustness and interpretability of our findings, we conducted repeated-measures ANOVAs across all behavioral tasks (see Supplementary material). This approach allowed us to evaluate changes over time (pre- to post-intervention) and potential interaction effects between time and intervention group. To address concerns about multiple comparisons and reduce the likelihood of false-positive findings, we applied Bonferroni corrections in all *post hoc* analyses. The results of the repeated-measures ANOVAs confirmed the findings reported in the primary analyses. Specifically, no significant changes were observed in delay discounting rates (*k*) or area under the curve (*AUC*) across time or between groups, and no significant interaction effects emerged. Similarly, in the social discounting task, while a significant main effect of Time suggested a general increase in generosity, no significant differences were found between intervention groups or in the interaction between Time and Group. For the Iowa Gambling Task (IGT), a significant main effect of Time indicated overall improvement in advantageous decision-making; however, there were no significant between-group differences or interaction effects. These supplementary analyses, which included Bonferroni-corrected post hoc tests, support the robustness of our original results and confirm that observed changes were not driven by specific intervention groups but may reflect broader, non-specific effects of participating in the study.

The correlation analysis revealed several interesting findings. There was a highly significant positive correlation between the AUC of the Delay and Social discounting tasks, indicating a potential interaction between delay and social decision-making processes, similar to previous studies (Rachlin and Jones, 2008; Wainwright et al., 2018). Moreover, correlations were identified between the AuC of discounting tasks and the Life Satisfaction Scale, suggesting that lower impulsivity and greater altruism contribute to overall life satisfaction. However, the IGT task did not correlate significantly with other behavioral tasks, like previous studies (He et al., 2016; Mejía et al., 2016; Munir, 2023), nor the Life Satisfaction Scale. Additionally, demographic variables did not correlate significantly with the behavioral tasks or among themselves. These results give us confidence that the variables were well-controlled in the study.

Some limitations and biases in this study pertain to the small sample size within each group. For future studies, we recommend incorporating incentives beyond extra credit in subjects, given our assumption that increased time spent receiving DBT skill training is advantageous, this strategy has the potential to enhance attention levels during evaluation and training. We also suggest incorporating DBT worksheets and comparing various training modalities, including face-to-face interventions. Identifying the most cost-effective approach for imparting these skills to a diverse population and assessing the potential of DBT skills in preventing mental illness and enhancing wellbeing are of utmost importance.

The correlation analysis underscored the interplay between delay and social decision-making and their association with life satisfaction. These findings offer valuable insights into the decision-making processes and their potential impact on life satisfaction. Therefore, we highly recommend incorporating supplementary self-report measures to assess life satisfaction in post-evaluations, rather than solely relying on pre-evaluation assessments, as we did. It is essential to incorporate the evaluation of other scales alongside behavioral tasks.

Future research should investigate the integration of cognitive biases and decision-making heuristics as mechanisms to “nudge” individuals, particularly college students, toward more prosocial and less impulsive behaviors. For instance, DBT skill training could facilitate changes in delay and social discounting rates while enhancing learning outcomes in tasks such as the Iowa Gambling Task (IGT). Moreover, future studies could examine whether targeted interventions focusing on empathy and compassion might amplify the observed effects of mindfulness and interpersonal effectiveness on prosocial behavior. DBT skills training has the potential to influence decision-making in real-life contexts, such as improving academic performance or fostering greater community engagement. Finally, employing longitudinal designs and larger, more diverse sample populations could provide a deeper understanding of the long-term effects of these skills on life satisfaction, mental health prevention, and overall wellbeing. Additionally, future research should explore the efficacy of teleintervention and hybrid delivery models to provide cost-effective and accessible DBT training.

In conclusion, while intriguing trends in discounting rates and Iowa Gambling Task (IGT) performances were observed among various groups before and after training, no statistically significant differences emerged either between groups or within individual groups. However, this study has identified that distress tolerance and interpersonal effectiveness skills have the potential to reduce impulsivity and enhance prosocial behavior. Furthermore, there is a need for additional investigation into the efficacy of brief teleintervention-based Dialectical Behavior Therapy (DBT) skills training, which could help reduce costs and increase accessibility to a broader population.

## Data Availability

The original contributions presented in the study are included in the article/Supplementary material, further inquiries can be directed to the corresponding author.

## References

[ref1] Acuff S. F. MacKillop J. Murphy J. G. (2023). A contextualized reinforcer pathology approach to addiction. Nat. Rev. Psychol. 2, 309–323. doi: 10.1038/s44159-023-00167-y, PMID: 37193018 PMC10028332

[ref2] American College Health Association (2012). American college health association-national college health assessment II: Reference group executive summary fall 2011. Hanover, MD: American College Health Association.

[ref3] Anokhin A. P. Golosheykin S. Mulligan R. C. (2015). Long-term test–retest reliability of delayed reward discounting in adolescents. Behav. Process. 111, 55–59. doi: 10.1016/j.beproc.2014.11.008, PMID: 25447508 PMC4306354

[ref4] Avila-Chauvet L. Cruz D. M. García-Leal Ó. Kluwe-Schiavon B. (2023). To produce or not to produce? Contrasting the effect of substance abuse in social decision-making situations. Heliyon 9:e19714. doi: 10.1016/j.heliyon.2023.e19714, PMID: 37809835 PMC10559002

[ref5] Barragán Torres L. González Vázquez J. Medina-Mora M. E. Ayala Velázquez H. (2005). Adaptación de un modelo de intervención cognoscitivo-conductual para usuarios dependientes de alcohol y otras drogas a población mexicana: Un estudio piloto. Salud Mental 28, 61–71.

[ref6] Bean C. A. Aurora P. Maddox C. J. Mekota R. Updegraff A. (2022). A comparison of telehealth versus in-person group therapy: results from a DBT-based dual diagnosis IOP. J. Clin. Psychol. 78, 2073–2086. doi: 10.1002/jclp.23374, PMID: 35531794 PMC9790325

[ref7] Bechara A. Damasio A. R. Damasio H. Anderson S. W. (1994). Insensitivity to future consequences following damage to human prefrontal cortex. Cognition 50, 7–15. doi: 10.1016/0010-0277(94)90018-3, PMID: 8039375

[ref8] Bickel W. K. Yi R. Landes R. D. Hill P. F. Baxter C. (2011). Remember the future: working memory training decreases delay discounting among stimulant addicts. Biol. Psychiatry 69, 260–265. doi: 10.1016/j.biopsych.2010.08.017, PMID: 20965498 PMC3015021

[ref9] Blanco C. Okuda M. Wright C. Hasin D. S. Grant B. F. Liu S. M.. (2008). Mental health of college students and their noncollege-attending peers: results from the National Epidemiologic Study on alcohol and related conditions. Arch. Gen. Psychiatry 65, 1429–1437. doi: 10.1001/archpsyc.65.12.1429, PMID: 19047530 PMC2734947

[ref10] Bornovalova M. A. Lejuez C. W. Daughters S. B. Rosenthal M. Z. Lynch T. R. (2005). Impulsivity as a common process across borderline personality and substance use disorders. Clin. Psychol. Rev. 25, 790–812. doi: 10.1016/j.cpr.2005.05.005, PMID: 16005556

[ref11] Bradstreet M. P. Higgins S. T. Heil S. H. Badger G. J. Skelly J. M. Lynch M. E.. (2012). Social discounting and cigarette smoking during pregnancy. J. Behav. Decis. Making 25, 502–511. doi: 10.1002/bdm.750 PMC3496264 23162211

[ref12] Buelow M. T. Barnhart W. R. (2018). Test–retest reliability of common behavioral decision making tasks. Arch. Clin. Neuropsychol. 33, 125–129. doi: 10.1093/arclin/acx038, PMID: 28430836

[ref13] Buelow M. T. Blaine A. L. (2015). The assessment of risky decision making: a factor analysis of performance on the Iowa gambling task, balloon analogue risk task, and Columbia card task. Psychol. Assess. 27, 777–785. doi: 10.1037/a0038622, PMID: 25580611

[ref14] Cavicchioli M. Movalli M. Bruni A. Terragni R. Maria Elena G. Borgia E.. (2023). The initial efficacy of stand-alone DBT skills training for treating impulsivity among individuals with alcohol and other substance use disorders. Behav. Ther. 54, 809–822. doi: 10.1016/j.beth.2023.02.006, PMID: 37597959

[ref15] Chan C. C. Alter S. Hazlett E. A. Shafritz K. M. Yehuda R. Goodman M.. (2023). Neural correlates of impulsivity in bipolar disorder: a systematic review and clinical implications. Neurosci. Biobehav. Rev. 105:109. doi: 10.1016/j.neubiorev.2023.105109 PMC11073484 36813146

[ref16] Chugani C. D. (2015). Dialectical behavior therapy in college counseling centers: current literature and implications for practice. J. Coll. Stud. Psychother. 29, 120–131. doi: 10.1080/87568225.2015.1008368

[ref17] Chugani C. D. (2017). Adapting dialectical behavior therapy for college counseling centers. J. Coll. Couns. 20, 67–80. doi: 10.1002/jocc.12059

[ref18] De Wilde B. Bechara A. Sabbe B. Hulstijn W. Dom G. (2013). Risky decision-making but not delay discounting improves during inpatient treatment of polysubstance-dependent alcoholics. Front. Psych. 4, 1–7. doi: 10.3389/fpsyt.2013.00091 PMC3760441 24027538

[ref19] Dennhardt A. A. Yurasek A. M. Murphy J. G. (2015). Change in delay discounting and substance reward value following a brief alcohol and drug use intervention. J. Exp. Anal. Behav. 103, 125–140. doi: 10.1002/jeab.121, PMID: 25533393

[ref20] He Q. Chen M. Chen C. Xue G. Feng T. Bechara A. (2016). Anodal stimulation of the left DLPFC increases IGT scores and decreases delay discounting rate in healthy males. Front. Psychol. 7:1421. doi: 10.3389/fpsyg.2016.01421, PMID: 27703440 PMC5028393

[ref21] Hernández J. Ortega M. González C. Díaz M. Téllez Z. Gutiérrez-Cardona C.. (2021). Evaluación de la efectividad del programa DBT STEPS-A en estudiantes universitarios mexicanos. Psicol. Salud 31, 103–112. doi: 10.25009/pys.v31i1.2680

[ref22] Jones C. (2013). Delay discounting rates, relapse, and treatment satisfaction in young adults. [Doctoral dissertation, Capella University]. Available online at: http://search.proquest.com/docview/1492338109

[ref23] Jones B. A. Rachlin H. (2009). Delay, probability, and social discounting in a public goods game. J. Exp. Anal. Behav. 91, 61–73. doi: 10.1901/jeab.2009.91-61, PMID: 19230512 PMC2614818

[ref24] Kluwe-Schiavon B. Kexel A. Manenti G. Cole D. M. Baumgartner M. R. Grassi-Oliveira R.. (2020). Sensitivity to gains during risky decision-making differentiates chronic cocaine users from stimulant-naïve controls. Behav. Brain Res. 379:112386. doi: 10.1016/j.bbr.2019.112386 31778734

[ref25] Landes R. D. Christensen D. R. Bickel W. K. (2012). Delay discounting decreases in those completing treatment for opioid dependence. Exp. Clin. Psychopharmacol. 20, 302–309. doi: 10.1037/a0027391, PMID: 22369670 PMC3972253

[ref26] Linehan M. (2015a). DBT® Skills Training Manual. 2nd Edn. New York, NY: Guilford Press. Available online at: https://www.guilford.com/books/DBT-Skills-Training-Manual/Marsha-Linehan/9781462516995?srsltid=AfmBOopeSK3MP2zvvMaLOL_42mBrpyW9nwibx1zw3AQC-okZmheirWny

[ref27] Linehan M. (2015b). DBT® Skills Training Handouts and Worksheets. 2nd Edn. New York, NY: Guilford Publications. Available online at: https://www.guilford.com/books/DBT-Skills-Training-Handouts-and-Worksheets/Marsha-Linehan/9781572307810

[ref28] López A. A. (2014). Examining delay discounting and response to incentive-based smoking-cessation treatment among pregnant women. [Doctoral dissertation, the University of Vermont]. Available online at: https://www.proquest.com/openview/4a35fcd4dd1584ae1e5f4c4caa143702/1?pq-origsite=gscholar&cbl=18750

[ref29] López-Torrecillas F. Nieto-Ruiz A. Velasco-Ortuño S. Lara-Fernández M. López-Quirantes E. M. Castillo-Fernández E. (2014). The role of impulsivity in dropout from treatment for cigarette smoking. Compr. Psychiatry 55, 1609–1613. doi: 10.1016/j.comppsych.2014.06.004, PMID: 25066693

[ref30] Mejía D. Avila-Chauvet L. Toledo-Fernández A. (2022). Decision-making under risk and uncertainty by substance abusers and healthy controls. Front. Psych. 12:788280. doi: 10.3389/fpsyt.2021.788280, PMID: 35153858 PMC8833085

[ref31] Mejía D. Morales-Chainé S. Nieto J. (2016). Efecto del tratamiento sobre el descuento temporal y probabilístico en participantes con trastorno por uso de crack. Rev. Int. Investig. Adicc. 2, 4–15. doi: 10.28931/riiad.2016.1.02

[ref32] Morrison K. Madden G. Odum A. Friedel J. Twohing M. (2014). Altering impulsive decision making with an acceptance-based procedure. Behav. Ther. 45, 630–639. doi: 10.1016/j.beth.2014.01.001 25022774 PMC5661961

[ref33] Muhomba M. Chugani C. D. Uliaszek A. A. Kannan D. (2017). Distress tolerance skills for college students: a pilot investigation of a brief DBT group skills training program. J. Coll. Stud. Psychother. 31, 247–256. doi: 10.1080/87568225.2017.1294469

[ref34] Munir S. (2023). Time is money: Using delay discounting and reflection to improve decision-making in the Iowa Gambling Task. [Doctoral dissertation, Hollins University]. Available online at: https://digitalcommons.hollins.edu/ughonors/58/

[ref35] Myerson J. Green L. Warusawitharana M. (2001). Area under the curve as a measure of discounting. J. Exp. Anal. Behav. 76, 235–243. doi: 10.1901/jeab.2001.76-235, PMID: 11599641 PMC1284836

[ref36] Pedrelli P. Nyer M. Yeung A. Zulauf C. Wilens T. (2015). College students: mental health problems and treatment considerations. Acad. Psychiatry 39, 503–511. doi: 10.1007/s40596-014-0205-9, PMID: 25142250 PMC4527955

[ref37] Rachlin H. Jones B. A. (2008). Social discounting and delay discounting. J. Behav. Decis. Making 21, 29–43. doi: 10.1002/bdm.567

[ref38] Secades-Villa R. Weidberg S. García-Rodríguez O. Fernández-Hermida J. R. Yoon J. H. (2014). Decreased delay discounting in former cigarette smokers at one year after treatment. Addict. Behav. 39, 1087–1093. doi: 10.1016/j.addbeh.2014.03.015, PMID: 24661901

[ref39] Sharp C. Barr G. Ross D. Bhimani R. Ha C. Vuchinich R. (2012). Social discounting and externalizing behavior problems in boys. J. Behav. Decis. Making 25, 239–247. doi: 10.1002/bdm.719

[ref40] Soler J. Elices M. Pascual J. C. Martín-Blanco A. Feliu-Soler A. Carmona C.. (2016). Effects of mindfulness training on different components of impulsivity in borderline personality disorder: results from a pilot randomized study. Borderline Personal. Disord. Emot. Dysregul. 3, 1–10. doi: 10.1186/s40479-015-0035-8, PMID: 26759718 PMC4709962

[ref41] Tuen Y. J. Bulley A. Palombo D. J. O'Connor B. B. (2023). Social value at a distance: higher identification with all of humanity is associated with reduced social discounting. Cognition 230:105283. doi: 10.1016/j.cognition.2022.105283, PMID: 36209687

[ref42] van Leeuwen H. Sinnaeve R. Witteveen U. Van Daele T. Ossewaarde L. Egger J. I.. (2021). Reviewing the availability, efficacy and clinical utility of telepsychology in dialectical behavior therapy (tele-DBT). Borderline Personal. Disord. Emot. Dysregul. 8, 1–15. doi: 10.1186/s40479-021-00165-7 34717772 PMC8556811

[ref43] Wainwright K. Green B. Romanowich P. (2018). The relationship between delay and social discounting, and body mass index in university students. Psychol. Rec. 68, 441–449. doi: 10.1007/s40732-018-0287-y

[ref44] Warnell K. R. Maniscalco S. Baker S. Yi R. Redcay E. (2019). Social and delay discounting in autism spectrum disorder. Autism Res. 12, 870–877. doi: 10.1002/aur.2085, PMID: 30816644 PMC6941783

[ref45] Weathers M. T. (2022). Mindful attention for reading and class (MARC): a DBT-informed group intervention for college students with attention-deficit/hyperactivity disorder. [Doctoral dissertation, Xavier University]. Available online at: http://rave.ohiolink.edu/etdc/view?acc_num=xupsy1649449747891909

[ref46] Weidberg S. Landes R. D. García-Rodríguez O. Yoon J. H. Secades-Villa R. (2015). Interaction effect of contingency management and sex on delay-discounting changes among treatment-seeking smokers. Exp. Clin. Psychopharmacol. 23, 361–368. doi: 10.1037/pha0000043, PMID: 26375514

[ref47] Weidel J. J. (2013). The relationship of temporal discounting and working alliance to substance abuse treatment process in Hispanic adolescents. [Doctoral dissertation, University of Miami]. Available online at: http://scholarlyrepository.miami.edu/oa_dissertations/1088

[ref48] Wilks C. R. Yin Q. Zuromski K. L. (2020). User experience affects dropout from internet-delivered dialectical behavior therapy. Telemed. e-Health 26, 794–797. doi: 10.1089/tmj.2019.0124, PMID: 31502945 PMC7301319

