# Supplementary material for: Shaping decision-making with screen time: video-based dialectical behavior therapy skills training for college students
Source: Front Psychol. 2025 Jul 9;16:1609744. doi: 10.3389/fpsyg.2025.1609744 (PMC12285534; doi:10.3389/fpsyg.2025.1609744)
Supplement: Supplementary file 1 [file Data_Sheet_1.pdf]

Supplementary material

Website

Pre-Test

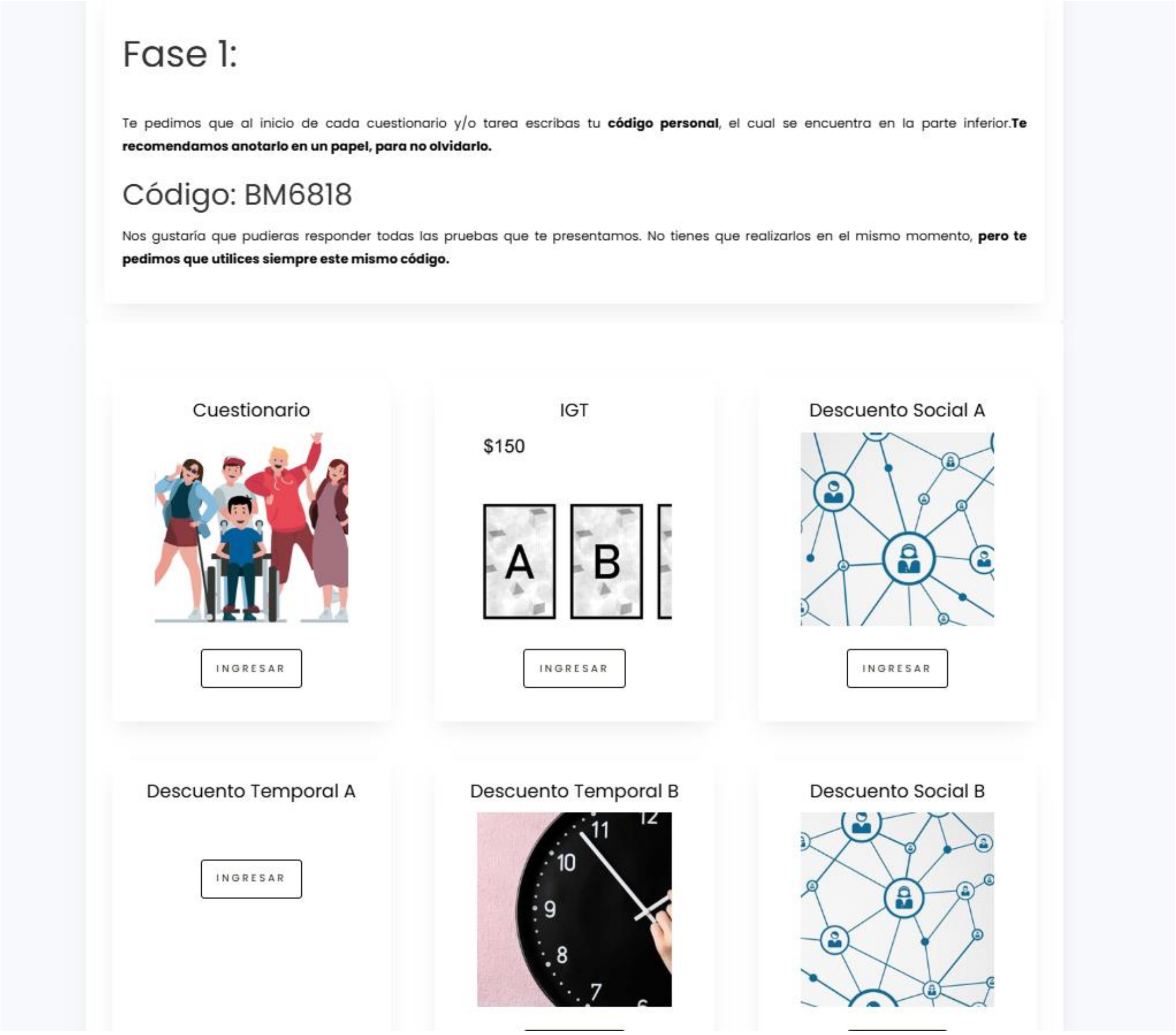

Video training

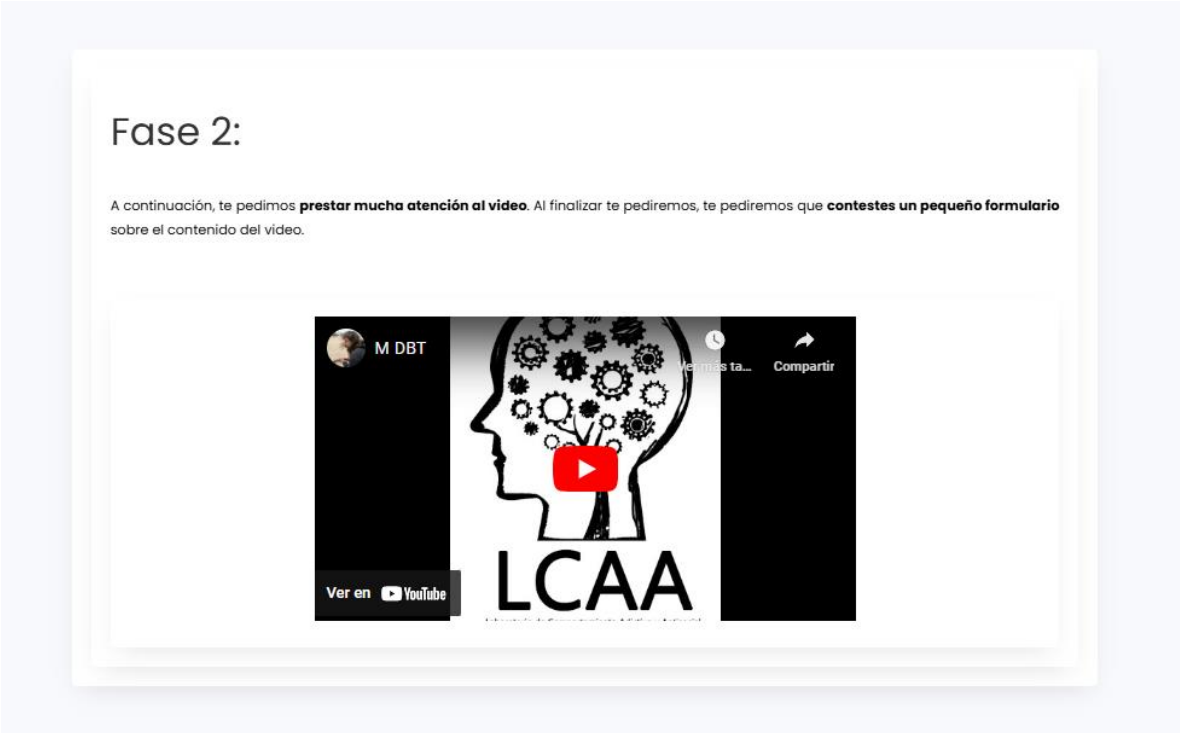

Post-Test

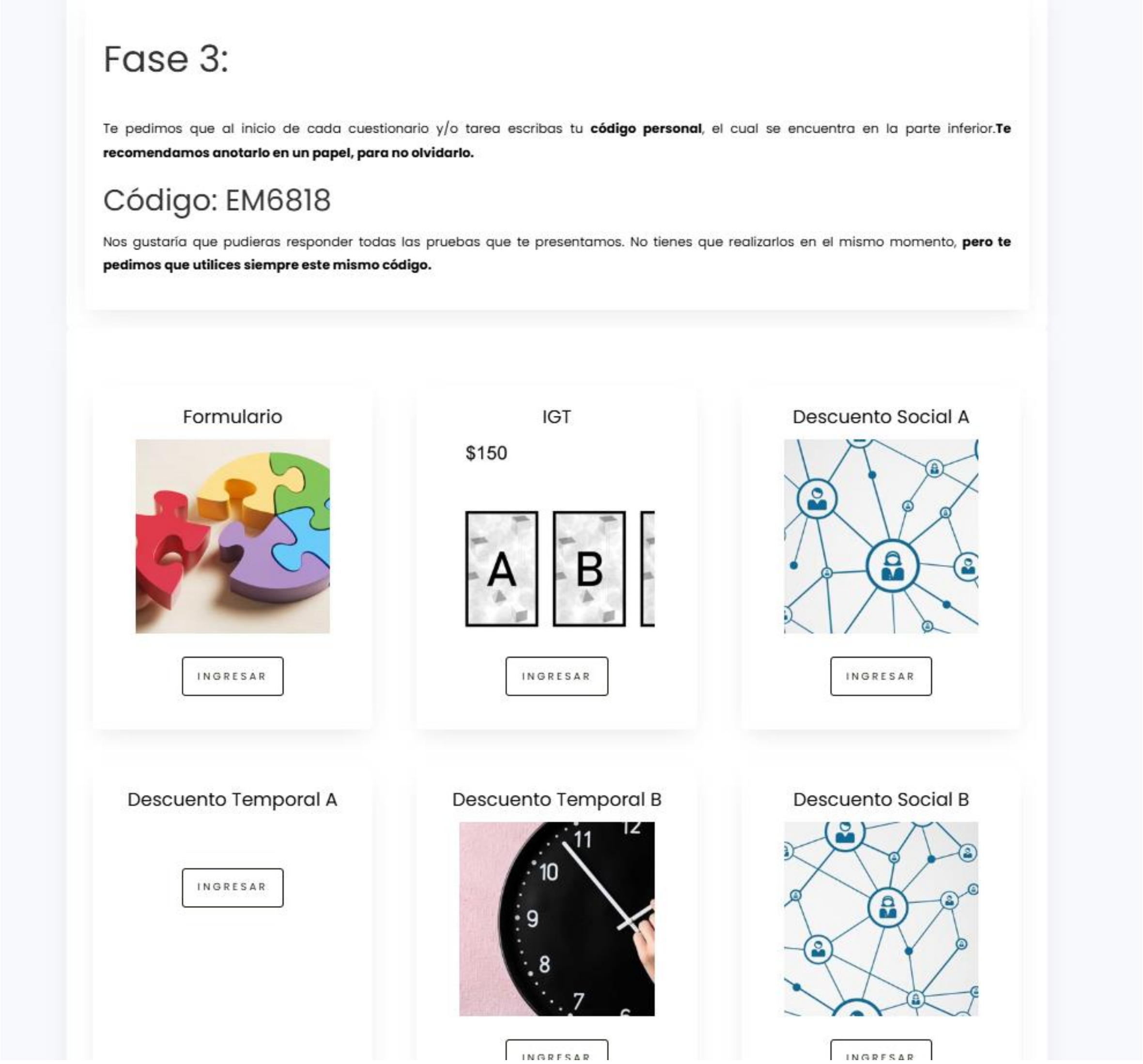
Fase 3:
Te pedimos que al inicio de cada cuestionario y/o tarea escribas tu código personal, el cual se encuentra en la parte inferior.Te recomendamos anotarlo en un papel, para no olvidarlo.
Código: EM6818
Nos gustaría que pudieras responder todas las pruebas que te presentamos. No tienes que realizarlos en el mismo momento, pero te pedimos que utilices siempre este mismo código.
Formulario
INGRESAR
IGT
$150
A
B
INGRESAR
Descuento Social A
INGRESAR
Descuento Temporal A
INGRESAR
Descuento Temporal B
Descuento Social B
