# Supplementary material for: Shaping decision-making with screen time: video-based dialectical behavior therapy skills training for college students
Source: Front Psychol. 2025 Jul 9;16:1609744. doi: 10.3389/fpsyg.2025.1609744 (PMC12285534; doi:10.3389/fpsyg.2025.1609744)
Supplement: Supplementary file 3 [file Data_Sheet_3.pdf]

Supplementary Analysis: Repeated-Measures ANOVA and Post Hoc Tests

Delay discounting task

To complement the primary analyses and address potential concerns about multiple comparisons, we conducted a repeated-measures ANOVA on the delay discounting rate (*k*), with Time (pre vs. post) as the within-subjects factor and Group (Control, Interpersonal Effectiveness, Mindfulness, Emotion Regulation, Distress Tolerance) as the between-subjects factor.

The analysis revealed no significant main effect of Time ($F(1, 90) = .527$, $p = .470$, $\eta^2 = .015$) nor a main effect of Group ($F(4, 90) = .691$, $p = .600$, $\eta^2 = .003$). Additionally, the interaction between Time and Group was not statistically significant ($F(4, 90) = .691$, $p = .600$, $\eta^2 = .015$). To further explore potential group differences, we conducted post hoc comparisons with Bonferroni correction. The results of these tests are consistent with the main manuscript findings, none of the pairwise comparisons reached statistical significance (all *p values* > .05). The differences in delay discounting rates (*k*) between any two groups, at either baseline or post-training, as well as the within-group pre-post changes, yielded negligible effect sizes and did not reach statistical significance.

In relation to the area under the curve (AUC) a repeated-measures ANOVA revealed no significant main effect of Time ($F(1, 90) = .428$, $p = .514$, $\eta^2 = .001$) and no significant Time and Group interaction ($F(4, 90) = .681$, $p = .607$, $\eta^2 = .001$). Although the between-groups effect approached statistical significance ($F(4, 90) = 1.963$, $p = .107$, $\eta^2 = .093$), it did not reach $p < .05$ threshold. Bonferroni-corrected post hoc comparisons showed no statistically significant differences between any pair of groups at either time point (all *p values* > .05).

Social discounting task

To examine changes in altruism-related decision-making, a repeated-measures ANOVA was conducted on social discounting, with Time (pre vs. post) as the within-subjects factor and Group (Control, Interpersonal Effectiveness, Mindfulness, Emotion Regulation, Distress Tolerance) as the between-subjects factor.

The analysis revealed a significant main effect of Time ($F(1, 90) = 6.633$, $p = .012$, $\eta^2 = .005$), indicating a general reduction in *k* values across participants, consistent with increased generosity or reduced discounting. However, the interaction effect between Time and Group was not statistically significant ($F(4, 90) = .751$, $p = .560$, $\eta^2 = .011$), nor was the main effect of Group ($F(4, 90) = .821$, $p = .515$, $\eta^2 = .030$). Bonferroni-adjusted post hoc comparisons showed no statistically significant differences between any groups (all *p* values > .05). Although a moderate effect size was observed in the post-intervention comparison between the Interpersonal Effectiveness and Emotion Regulation groups ($d = –0.885$).

In relation to the area under the curve (AUC) from the social discounting task as the dependent variable, a repeated-measures ANOVA revealed no significant main effect of Time ($F(1, 90) = .983$, $p = .324$,$\eta^2 = .008$) and no significant Time and Group interaction ($F(4, 90) = 1.703$, $p = .156$, $\eta^2 = .001$). Although the between-groups effect approached statistical significance ($F(4, 90) = 1.956$, $p = .108$, $\eta^2 = .070$) it did not reach $p < .05$ threshold. Bonferroni-corrected post hoc comparisons showed no statistically significant differences between any pair of groups at either time point (all *p values* > .05).

Results of Iowa Gambling Test

Regarding the proportion of advantageous choices in the Iowa Gambling Task (IGT), a repeated-measures ANOVA revealed a significant main effect of Time ($F(1, 90) = 5.813$, $p = .018$, $\eta^2 = .016$), indicating a general improvement in advantageous decision-making

following the intervention. However, the Time and Group interaction was not significant ($F(4, 90) = 1.166$, $p = .331$, $\eta^2 = .013$), nor was the main effect of Group ($F(4, 90) = 1.571$, $p = .189$, $\eta^2 = .047$). Bonferroni-corrected post hoc comparisons showed no statistically significant differences between any pair of groups at either time point (all *p values* > .05).
